# Supplementary material for: Spatiotemporal Mislocalization of Nuclear Membrane-Associated Proteins in γ-Irradiation-Induced Senescent Cells
Source: Cells. 2020 Apr 17;9(4):999. doi: 10.3390/cells9040999 (PMC7227243; doi:10.3390/cells9040999)
Supplement: Supplementary file 1 [file cells-09-00999-s001.pdf]

Figure S1 – Svobodova Kovarikova et al.

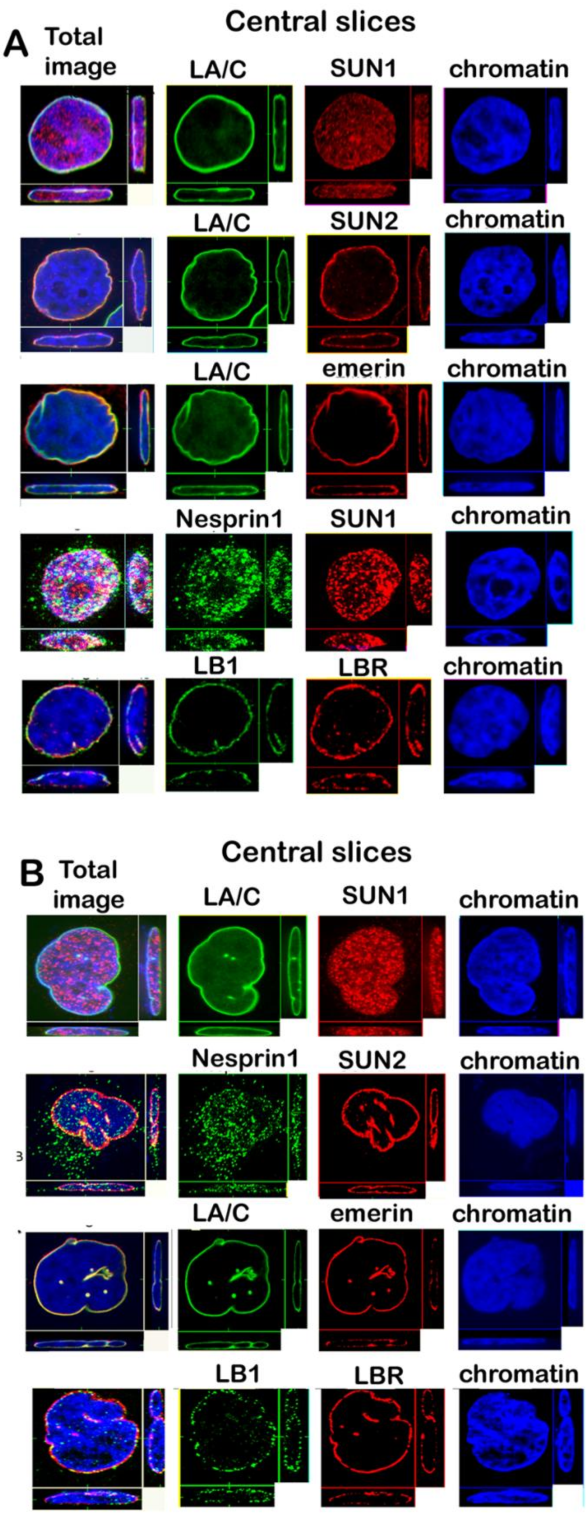

Fig. S1A, B Localization of LINC complex proteins (Nesprin-1, SUN1 and SUN2), emerin, LAC, LBR and LB1 in control non-irradiated (A) MCF7 and (B) U2OS cells. The same localization of these proteins is also in control cells of non-irradiated MCF7-(LBR(-) and U2OS-(LBR(-) cells (not shown). Size bar indicates 5 µm.

Figure 2A-C Svobodova Kovarikova et al.

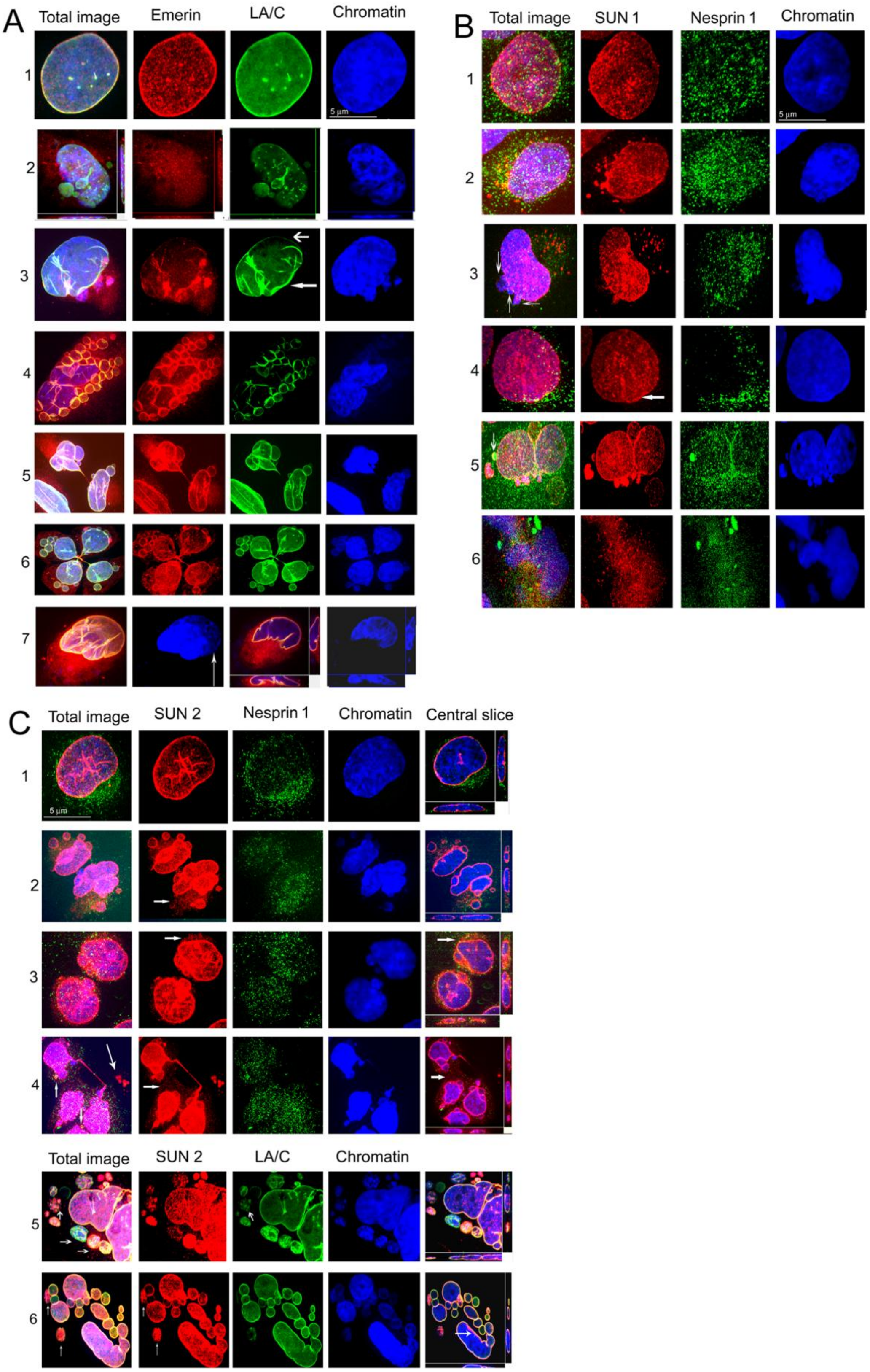

Figure 2A-C - continued

Fig. S2A. Examples of emerin and LA/C mislocalization MCF7-LBR(-) and U2OS-(LBR(-)cells 24 h PI with 8 Gy of γ-rays. 1 – Control U2OS-LBR(-) nucleus, **2** – U2OS-LBR(-) with blisters and partially fractionated LA/C in the NE, MN and blisters; emerin is dispersed in cytoplasm. **3** – U2OS-LBR(-) with reinforced LA and emerin (long arrow) at a region of NE and very thin layer of both proteins at upper part of the nucleus (short arrow). Emerin is dispersed into cytoplasm and concentrated in the NM at its reinforced layer**. 4** – Fragmented MCF7-LBR(-) nucleus. All MN are bordered by LA/C (green) and emerin (red) that is also inside MN and partially dispersed around this complex. **5** – 3 nuclei of MCF7-LBR(-) cell. The nucleus located in the center of the image has emerin dispersed to cytosol and a MN; a nucleus at the top of the picture is convoluted. The nucleus in the left corner has reinforced layer of LA/C. **6** –2 ultrafine anaphase bridges separating 2 pairs of daughter nuclei of MCF7-LBR(-) cell. All nuclei have several MN and 1MN has emerin polar caps; emerin forms also small dots around nuclei**. 7**- Honeycombing nucleus of U2OS-LBR(-) cell is losing heterochromatin (arrow) and disperse emerin. Size bar indicates 5 µm

Fig. S2B. Examples of SUN1 and nesprin 1 mislocalization in MCF7-LBR(-) and U2OS-LBR(-) 24 h PI with 8 Gy of γ-rays. 1 – U2OS-LBR(-) control. **2** – MCF7-LBR(-) has SUN1 amplified at a region of NM, where this protein forms small clumps liberated to the cytoplasm. High density of nesprin 1 dots is concentrated in the space where SUN1 is liberated to the cytosol. **3** - MCF7-LBR(-) containing 3 MN attached to the nucleus (arrows) and SUN1 amplified in a region of the nucleus, where clumps of SUN1 are liberated to cytoplasm. A group of the small clumps is surrounded by a cloud of nesprin 1 dots. **4** – A giant nucleus of U2OS-LBR(-) containing SUN1, amplified in a region of the nucleus (arrow). At this side, the nucleus is surrounded by many dots of nesprin-1**. 5 -** Convoluted nucleus of U2OS-LBR(-) contains several MN; Micronuclei containing HC are filled with amplified SUN1 and one MN contains also nesprin1 (arrow). 2 large nuclei are located close to each other and have a reinforced common layer of SUN1 at this sit. **6** – Convoluted and blistered nucleus of U2OS-LBR(-) lost completely the NE, it has dissipated SUN1 together with nesprins-1 in the cytoplasm. Sun 1 form a large cloud of tiny and larger dots combined with tiny dots of nesprin-1. This protein, forms large clumps at some regions of the nucleus (green clumps). Condensed chromatin has a convoluted form. Size bar indicates 5 µm.

Fig. S2C Examples of SUN2 and nesprin 1 as well as SUN2 and LA/C mislocalization in MCF-LBR(-) and U2OS-LBR(-) 24 h PI with the dose of 8 Gy of γ-rays 1- a control U2OS-LBR(-) with visualized SUN2 and nesprin-1. **2** –A convoluted nuclei of U2OS-LBR(-) containing several MN with EU, bordered or filed with SUN2. SUN 2 is reinforced in the parts of nuclei that are partially disintegrated into small dots forming a layer around of the nuclei or even clouds of dense dots of SUN2 (arrow). Nesprin usually accompany these clouds of small points of SUN2. **3** – Convoluted nuclei of MCF7-LBR(-) have also reinforced layer of SUN2 forming a layer of tiny dots (arrow) around a nuclear membrane (arrow). **4** - A group of nuclei connected with anaphase bridges. Each nucleus has several MN attached to the nuclei. The MN contain HC and are filled with SUN2. SUN 2 is amplified in all MN and is also disintegrated into dense tiny dots (arrows). In the proximity of the anaphase bridge, several larger fragment of SUN2 can be seen (long arrow). Nesprin-1 accompanies these convoluted nuclei especially at the places where SUN2 forms tiny dots – **5** Large fragmented nucleus of an U2OS-LBR(-) cell with many MN containing HC presents a half of twins connected with an anaphase bridge. MN are bordered by LAC similarly as the nucleus. Several MN are filled with SUN2 and fragmented LAC; in some nuclei, both SUN2 and LA/C are fragmented (wide arrows). In addition, there are small dots of fragmented SUN 2 in the cytoplasm (arrows). **6** – Similar image as is the disintegrated nucleus of MCF7-LBR(-), are all nuclei and MN bordered by LA/C and a majority is filled with SUN2; some of them are filled also with amplified LA/C and SUN2. In addition, there are also 2 large clumps of SUN2 (small arrows). The nucleus has reinforced layer of SUN 2 at a part of NE (arrow) and in a MN. Size bar indicates 5µm.

Figure S3 – Svobodova Kovarikova et al.

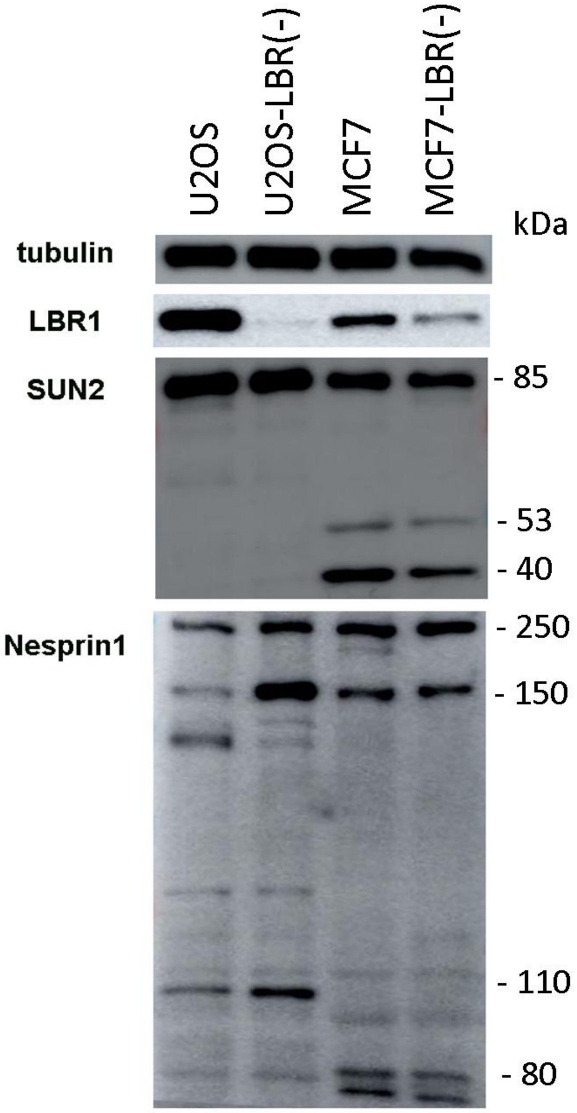

Fig. S3. Western blot analysis of LBR, SUN2 and nesprin-1 expression in control and LBR-deficient MCF7 and U2OS cells. Note, weak or missing signals in MCF7-LBR(-) and U2OS-(LBR(-) cells after the staining of blots with an antibody against LBR.

Table S1A, B. Frequencies of nuclear morphology defects induced in MCF7, and U2OS cells before irradiation and in different time after irradiation with the dose of 8 Gy of γ-rays. (A) MCF7, (B) U2OS. Over 100 cells were counted in each group in 3 independent experiments and the mean number of cells/experiment ± SE are presented at each time PI. Number of cells containing a specific defect is expressed as a percentage of mean number of cells ± SE. P values were calculated using the student t-test relatively to control unirradiated cells. (*p<0,05). The values, which are presented graphically in Fig. 1.A,B are marked in yellow.

Cell characteristics				Defects in nuclear morphology							
MCF7 Cell line **A**	Cell number Mean	LBR/LB1 Reduction	Cells without changes	Cells with MN	Blister	Convo-volute	Anaph bridge	Honey-combing	Gigant nuclei	Wrinkle	Fragments
	Cell number %										
control	134± 1,471	0	94,2± 0,616	6,0 0,646	--	-------	---	-----	---		----
24h PI	187 ±1,379	55/45± 0,772/ 1,023	38,2± 0,496	33,5 ± 1,21	17,9 ± 0,391	32,7± 1,755	4,5± 0,5	----	--	--	2,4± 1,95
72h PI	158± 1,021	91/80± 0,697/ 0,404	1	83,7 2,513	26,5± 0,521	41,8± 1,128	10.9± 0,923	--	--	14,1± 0,965	16,2± 1,16
7D PI	222± 1,157	99/90± 0,660/ 0,660	0	82± 1,191	57,6± 1,571	44,4± 1,192	7,1± 0,52	--	--	6,2± 0,55	18,8± 1,55
U2OS Cell line **B**						--					--
control	104± 0,678	0	89,0± 1,414	7,3 0,582	---	--	--	1,2± 0,091	--		--
24h PI	112± 0,711	60/66± 1.174/ 0,637	40,1± 0,544	33,0± 1,411	22,5± 1,021	16,9± 0,331	--	10,0± 0,552	28± 1,412	--	--
72h PI	113 ±0,509	69/70± 0,374/ 0,326	0	65.9± 0,651	55,6± 0,965	63± 2,016	--	11,0± 0,98	10,5± 1,042	10,6± 1,07	15± 1,037
7D PI	76± 0,852	82/99± 1,055/ 0,346	0	71± 1,521	63,4± 2,482	71,0± 6,92	--	5,20± 0,17	39,1± 1,721	5,20± 0,05	47,0± 4,05

Table S1C, D. Frequencies of nuclear morphology defects induced in MCF7-LBR(-), and U2OS-LBR(-) before irradiation and at different time after irradiation with the dose of 8 Gy of γ-rays. (A) MCF7-LBR(-), (B) U2OS-(LBR(-) cells. Over 100 cells were counted in each group in 3 independent experiments and the mean number of cells/experiment ± SE are presented in control and irradiated cells at 27h PI and 7 D PI. Number of cells containing specific defect is expressed as a percentage of mean no. of cells ± SE . P values were calculated using the student t-test relatively to control non-irradiated cells. (*p<0,05). The values, which are represented graphically in Fig. 1.A,B, are marked in yellow.

Cell characteristics			Defects in nuclear morphology						
**MCF7-LBR(-) C**	Cell Number Mean	LBR/LB1 reduced	Cells With MN	Blisters	Convoluted cells	Anaph bridges	Honey combing cells	Giant cells	Frag mented cells
		**Cell number %**							
**Control**	163±<br>1,732	80/74 ±<br>1,471/<br>0,816	3,6 ±<br>0,186	0,6±<br>0,032	---	---	---	---	----
**8Gy/24h PI**	268±<br>2,445	86/82<br>1,08/<br>2,828	56,8±<br>1,303	23,2±<br>0,257	49,9±<br>1,210	17,1±<br>0,876	-----	-----	1,4±<br>0,326
**8Gy/7D PI**	126±<br>1,2715	97/95±<br>0,905/<br>0,658	89,5±<br>1,458<br>5	61,9 ±<br>1,210	94,5 ±<br>1,3025	17,5 ±<br>1,0085	----	---	13,57<br>0,4755
**U2OS-LBR(-) D**									
**Control**	135±<br>2,073	72/72<br>±0,408	1,8±<br>0,039<br>7	0,61±<br>0,0460	----	----	0,61±<br>0,460	---	---
**8Gy/24h PI**	154±<br>2,014	86/89±<br>1,914/<br>1,796	40,1±<br>0,489	32,6±<br>0,489	41,2 ±<br>0,941	1,2 ±<br>0,0288	2,8±<br>0,106	27,8±<br>0,432	1.8±<br>0,0244
**8Gy/7D PI**	95±<br>1,632	99/99±<br>1,914/<br>1,542	74,2±<br>0.326	65,9 ±<br>0,535	82,4±<br>0,432	1,0 ±<br>0,080	1,2 ±<br>0.256	46,9 ±<br>0,660	40.2±<br>0.439

Table S2A Frequencies of mislocalization of LINC complex proteins in MCF7 cells before irradiation and at 24h, 72h and 7 D PI with the dose of 8 Gy of γ-rays

PROTEIN	LOCATION	Control	24 h PI	72 h PI	7D PI
		Cells with defect %			
Emerin					
	Clumps in cytosol	4,1±0,535			
SUN1					
	Clumps in cytosol	3,0±0,454			
SUN2					
	Clumps in cytosol	2,7±0,616			
Nesprin 1					
	Clumps in cytosol	2,5±0,244			
Emerin					
	Dispersion in cytosol		100±2,16		
	Clumps in cytosol		23±0,757	0	45,0±1,721
	Presence in MN		22,9±0,143	37,0±,0697	0
	Polar caps		0	0	13,6±0,408
Emerin+ LAC					
	Clumps in cytosol		16±0,143	31,0±2,16	4.5±0,516
	Presence in MN		14,5±0,112	24,9±0,648	40,5±0,946
LAC					
	Minor clumps in cytosol		6,2±0,509	6,5±0,244	0
	Presence in MN		2,5±0,712	2,5±0,694	0
SUN1					
	Clumps in cytosol		22±1,368	7,1±0,469	0
	Presence in MN		0	35,6±0,432	49,9±0,8426
	Polar caps		5,5±0,230	0	0
SUN1+Nesprin1					
	Presence in MN		0	50,0±1,414	40,8±0,969
Nesprin 1					
	Clumps in cytosol		16,5±0,509	5,9±0,828	23,8±0,535
	Polar caps		11,0±0,883	0	0
SUN2					
	Clumps in cytosol		10,2±0,714	0	36,0±0,529
	Presence in MN		35,0±0,535	58,9±1,699	0
SUN2+ Nesprin 1					
	Presence in MN		2,5±0,569	0	46,0±0,976

More than 100 cells were counted to detect location of SUN1, SUN2, nesprin1, emerin, LA/C in each group of 3 independent experiments. The results are presented as a percentage from a mean no. of cells ± SE. P values were calculated using the student t-test relatively to control unirradiated cells. (*p<0,05). The columns of values, which are represented graphically in Fig. 4. A are marked in yellow.

Table S2B

Frequencies of mislocalizations of LINC proteins before and after irradiation with the dose of 8 Gy of γ-rays in U2OS					
**PROTEIN**	**LOCATION**	**CONTROL**	**24 h PI**	**72 h PI**	**7D PI**
		**Cells with defect %**			
Emerin					
	Clumps in cytosol	2,8±0,346			
SUN1					
	Clumps in cytosol	4,0±0,560			
SUN2					
	Clumps in cytosol	2,0±0,580			
SUN2+ Nesprin 1					
	Polar caps	2,7±0,355			
Nesprin 1					
	Clumps in cytosol	3,0±0,216			
Emerin					
	Dispersion in cytosol		100±0,816		
	Polar caps		7, 0±0,294	25±0,197	16,0±0,697
	Clumps in cytosol		26,0±0,571	0	0
	Presence in MN	--	0	4,0±0,331	38,0±0,968
Emerin+ LA/C	Dispersion in cytosol		100±0,816		
	Polar caps	--	0	8,3±0,424	12,9±0,565
	Clumps in cytosol	--	0	12,9±0,374	0
	Presence in MN		0	12,4±0,374	0
SUN1					
	Clumps in cytosol	--	27,0±1.07	80.0±1,131	49,0±1,584
	Presence in MN	--	4,0±0,28	0	0
SUN1+ Nesprin 1					
	Clumps in cytosol	--	0	75,0±0,588	0
Nesprin 1					
	Clumps in cytosol	--	10,5±0,326	10,5±0,577	13,8±0,571
	Presence in MN	--	0	5,5±0,086	0
SUN2					
	Clumps in cytosol	--	4,9±0,668	30,0±1,042	0
	Presence in MN	--	0	11,0±0,424	56,0±0,294
SUN2+Nesprin1					
	Polar caps	--	4,4±0,535	0	0
	Clumps in cytosol				

More than 100 cells were counted to detect location of SUN1, SUN2, nesprin1, emerin, LA/C, in each group of 3 independent experiments. The results are presented as a percentage from a mean no. of cells ± SE. P values were calculated using the student t-test relatively to control unirradiated cells. (*p<0, 05). The columns of values, which are represented graphically in Fig. 4.B are bordered in yellow.

Table. S2C Frequencies of mislocalizations of LINC proteins before and after irradiation with 8 Gy of γ-rays in MCF7-LBR(-) cells

Protein	Location	Control	24 h PI	7 D PI
		Cells with defects %		
Emerin				
	Polar caps	2±0,268		
	Amplification at NM	2±0,268		
SUN2	cytosol	2,7±0,509		
Emerin		---		
	Dispersion in cytosol		100±3,295	
	Polar caps	--	12,0±0,571	
	MN	--	26,0±0,648	63,0±0,565
	Anaph bridge amplific	--	1,0±0,577	
	Large clumps in cytosol	--	40,0±0,697	16,0±0,993
Emerin + LAC				
	Clumps in cytosol	--	3,0±0,883	31,0±0,941
	MN		17,0±0,697	90,0±1,02
LA/C		--		
	MN		0	6,0±0,446
	Anaphase bridge amplif		2,0±0,675	
SUN 1				
	Amplification at NE and fragmentation in cytosol	--	31,4±1,070	9,0±0,787
	MN	--	55,0±1,398	100,0±3,559
	MN	--	17,4±0,166	18,0±0,355
SUN2				
	Large clumps in cytosol	--	8,0±0,698	12,0±0,653
	Polar caps	--	5,0±0,645	12,0±0,633
	MN	--	29,3±0,496	99,0±0,976
	Amplification at a part of NE+ fragmentation	--	3,08±0,365	3,0±0,509
	Larger clumps in cytosol	--	1.5±0,785	30,0±1,39
	MN	--	6,6±0,509	30,0±0,785
	Amplification of SUN2 at a part of NE			3,0±0,454
Nesprin 1				
	MN	--	8,5±0,286	12,0±0,637
	Clumps in cytosol	--	11,8±1,251	12,0±0,816
	NE location		5,7±0,326	27,0±0,637

More than 100 cells were counted to detect location of SUN1, SUN2, nesprin1, emerin, LA/C, in each group of 3 independent experiments. The results are presented as a percentage from a mean no. of cells ± SE. P values were calculated using the student t-test relatively to control unirradiated cells. (*p<0,05). The columns of values, which are represented graphically in Fig. 4 A are bordered in yellow.

Table S2D - Frequencies of mislocalizations of LINC proteins in U2OS-LBR(-) cells before and after irradiation with the dose of 8 Gy of γ-rays				
		Cells with defects %		
PROTEIN	LOCATION	Control	24 hPI	7DPI
Emerin				
	Clumps in cytosol	4,0±0,163		
SUN 1				
	Minor clumps in cytosol	6,2±0,216		
	Presence in MN	3,1±0,355		
SUN2	Minor clumps in cytosol	2,7±0,251		
SUN2+ nesprin 1	Polar caps	2,5±0,141		
LBR		----		
	Presence in MN	--	6,1±0,374	18,0±0,844
LB1				
	Small clumps in cytosol and at NM	--	11,0±,0556	26,1±0,804
LAC				
	Polar caps			3,0±0,668
	Irregularly amplified at NM	---	15,0±0,294	0
Emerin				
	Dispersion in cytosol		100±1,154	
	Presence in MN		29,6±0,576	21,1±0,730
	Clumps in cytosol		27,0±0,294	15,3±0,642
	Polar caps		13,5±0,496	0
	Fragmented emerin at NM		2,7±0,293	0
	Amplification on a part of NM or MN			9,2±0,355
Emerin+ LA/C				
	Amplification at NM		14,8±0,356	15,0±0,989
	Clumps in cytosol		13,5±0,424	
	Polar caps		5,4±0,374	
	Presence in MN			15,0±0,778
SUN1				
	Amplif and disintegration at NM		18,7±0,697	4,0±0,637
	Disintegration in cytosol		47,1±0,648	12,1±0,559
	Amplification at NM		8,3±0,326	
	Presence in MN		2,8±0,428	40,5±0,454
SUN2				
	Clumps in cytosol		53,4±0,408	32,0±0,697
	Presence in MN		33,4±1,821	12,0±0,619
	Amplif + desintegrationat NM		53,2±0,711	
Nesprin 1				
	Clumps in cytosol		24,0±0,637	48,0±0,683
SUN2+nesprins 1				
	Clumps in cytosol			44,0±0,588
	Polar caps			4,0±0,665
	Presence in MN			20,0±0,454

More than 100 cells were counted to detect location of SUN1, SUN2, nesprin1, emerin, LA/C, in each group of 3 independent experiments. The results are presented as a percentage from a mean no. of cells ± SE. P values were calculated using the student t-test relatively to control unirradiated cells. (*p<0,05). The columns of values, which are represented graphically in Fig. 4 B are bordered in yellow.
